# Tree component biomass expansion factors and root-to-shoot ratio of Lebombo ironwood: measurement uncertainty

**DOI:** 10.1186/s13021-015-0019-4

**Published:** 2015-04-12

**Authors:** Tarquinio Mateus Magalhães, Thomas Seifert

**Affiliations:** 1 Departamento de Engenharia Florestal, Universidade Eduardo Mondlane, Campus Universitário Principal, Edifício no 1, Maputo, Mozambique; 2 Department of Forest and Wood Science, University of Stellenbosch, Private Bag X1 Matieland, 7602 Stellenbosch, South Africa

**Keywords:** *Androstachys johnsonii* Prain, Mecrusse, Additivity, Belowground biomass, Forest inventory

## Abstract

**Background:**

National and regional aboveground biomass (AGB) estimates are generally computed based on standing stem volume estimates from forest inventories and default biomass expansion factors (BEFs). AGB estimates are converted to estimates of belowground biomass (BGB) using default root-to-shoot ratios (R/S). Thus, BEFs and R/S are not estimated in ordinary forest inventories, which results in uncertainty in estimates of AGB and BGB. Here, we measured BEF and R/S values (including uncertainty) for different components of Lebombo ironwood (*Androstachys johnsonii* Prain) trees and assessed their dependence on tree size.

**Results:**

The BEF values of tree components were unrelated or weakly related to tree size, and R/S was independent of tree size. BEF values varied from 0.02 for foliage to 1.31 Mg m^−3^ for whole tree; measurement uncertainty (SE) varied from 2.9% for stem BEF to 10.6% for whole-tree BEF. The belowground, aboveground, and whole-tree BEF-based biomass densities were 30 ± 2.3 (SE = 3.89%), 121 ± 7.84 (SE = 3.23%), and 151 ± 9.87 Mg ha^−1^(SE = 3.27%), respectively. R/S was 0.24 with an uncertainty of 3.4%.

**Conclusions:**

Based on the finding of independence or weak dependence of BEF on tree size, we concluded that, for *A. johnsonii*, constant component BEF values can be accurately used within the interval of harvested tree sizes.

## Background

National and regional aboveground biomass (AGB) estimates are generally calculated based on estimates of standing stem volume from forest inventories and from default biomass expansion factors (BEFs). The AGB estimates are converted into belowground biomass (BGB) using default root-to-shoot ratio (R/S) values. This method is commonly used to estimate carbon stocks for national greenhouse gas (GHG) inventories [1].

However, BEF and R/S values can vary according to vegetation type, precipitation regime, mean annual temperature [2], and tree age and size [3-7]; thus, use of default values for national- or regional-scale estimates might result in unreliable assessments of biomass, carbon, and GHGs. In addition, because BEF and R/S values are not estimated during ordinary forest inventories, uncertainty in estimates of AGB and BGB is mainly attributed to these parameters [8], and it thus represents a major gap in carbon accounting at regional and national levels [9]. Few studies have provided estimates of BEF and R/S with measures of uncertainty, and although individual R/S values for specific forest and woodland types have not been widely studied, these values enable more-accurate estimates of belowground biomass [2] when compared to default ones. Therefore, estimates of BEF and R/S with uncertainty are needed for different types of woodlands.

The objective of this study was to develop tree component BEF and R/S values with known uncertainty for *A. johnsonii.*

## Results

### Descriptive statistics of the collected data

**Figure 1:**
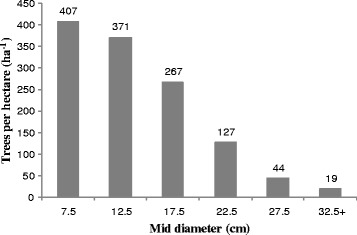
Diameter distribution histogram of phase-1 sampled *A. johnsonii* trees.

The number of trees recorded during the first sampling phase ranged from approximately 500 to >1000 ha^−1^ with an average of 1236 ha^−1^, distributed in each diameter class as shown in the Figure 1 – diameter distribution histogram – which follows a pattern of an inverse J-shaped curve, typical of an uneven-aged forest. The size and volume of the trees varied substantially (Table 1). The average AGB per tree $\left({\overline{w}}_{Shoo{t}_1}\right)$ was 97.95 kg. The dry weight of the components measured destructively during the second sampling phase, as well as Hohenadl form factor and stem volume, also varied considerably (Table 2).

**Table 1:** **Values of variables estimated for 3574** ***Androstachys johnsonii*** **trees in 23 plots during the first sampling phase**

**Statistic**	**Trees (ha** ^**-1**^ **)**	**Stem volume (m** ^**3**^ **)**	**Stem volume (m** ^**3**^ **/ha)**	**DBH (cm)**	**TH (m)**	**H (m)**
Average	1236.2187	0.0933	115.3149	13.4112	10.5616	10.4272
Minimum	541.1268	0.0020	66.8999	5.0000	1.8000	1.6000
Maximum	2220.2115	1.6463	170.4468	50.0000	22.5000	22.3000
SD	476.7204	0.1153	25.4407	6.2879	2.7637	2.7901
CV	38.5628	123.6853	22.0900	46.8856	26.1678	26.7583
SE	99.4031	0.0019	5.3048	0.1052	0.0462	0.0467
SE (%)	8.0409	2.0689	4.6061	0.7843	0.4377	0.4476

DBH, diameter at breast height; TH, total height; H, stem height; SD, standard deviation; CV, coefficient of variation; SE, standard error.

**Table 2:** **Values of variables for 93** ***Androstachys johnsonii*** **trees (a subset of the trees from phase 1) obtained during the second phase using destructive sampling**

	**Second phase variables**	**Average**	**Minimum**	**Maximum**	**SD**	**CV**
Component dry weight (kg)	Taproot + stump	23.651	1.474	71.926	18.926	80.019
	Lateral roots	24.083	0.746	100.815	23.945	99.428
	**Root system**	**47.735**	**2.545**	**162.105**	**41.210**	**86.331**
	Stem wood	124.068	4.947	357.348	99.497	80.196
	Stem bark	14.198	0.677	55.805	12.372	87.138
	**Stem**	**138.267**	**5.636**	**413.153**	**110.577**	**79.974**
	Branches	55.586	2.583	211.320	57.355	103.183
	Foliage	2.807	0.333	15.100	2.493	88.818
	**Crown**	**58.393**	**3.038**	**216.695**	**59.077**	**101.172**
	**Shoot system**	**196.659**	**9.823**	**590.863**	**163.713**	**83.247**
	**Total tree**	**244.394**	**12.484**	**752.571**	**204.330**	**83.607**
	Diameter at breast height (DBH) (cm)	17.5860	5.0000	32.0000	7.5122	42.7167
	Total height (TH) (m)	12.3230	5.0000	16.0000	2.1381	17.3508
Dendrometric variables	Stem length (H) (m)	10.7470	4.2500	14.8400	2.1381	22.7562
	Stem volume (v2) (m3)	0.1890	4.2500	0.5806	0.1512	22.7562
	Hohenadal form factor (fh)	0.4460	0.3002	0.6128	0.0592	13.2716

The major components and their values are indicated in bold font. SD, standard deviation; CV, coefficient of variation.

### Biomass expansion factors

The total tree and aboveground BEFs were approximately 131% and 105% of the stem volume, respectively (Table 3). For the major components, the stem had the highest BEF, and this value was more than two-fold higher than the BEF of crowns and roots. The standard error of all estimates was <11%; stem estimates were the most precise, and foliage estimates had the largest error (Table 3).

**Table 3:** **Component biomass expansion factors (BEF**_**h**_ **), their variances (VAR**_**BEF**_ **), standard errors (SE), and 95% confidence intervals (CI) for** ***Androstachys johnsonii*** **trees**

**#**	**Tree component**	**BEF**_**h**_ **(Mg m** ^**–3**^ **)**	**VAR**_**BEF**_ **(Mg** ^**2**^ **m** ^**–6**^ **)**	**SE (Mg m** ^**–3**^ **)**	**SE (%)**	**95% CI (Mg m** ^**–3**^ **)**	**95% CI (%)**
1	Taproot + stump	0.1407	3.6E-05	0.0060	4.2382	± 0.0119	±8.4764
2	Lateral roots	0.1162	4.4E-05	0.0067	5.7232	±0.0133	±11.4465
**3**	**Root system (1 + 2)**	**0.2569**	**1.0E-04**	**0.0100**	**3.8930**	**±0.0200**	**±7.7860**
4	Stem wood	0.6569	3.6E-04	0.0191	2.9046	±0.0382	±5.8092
5	Stem bark	0.0765	1.3E-05	0.0036	4.7534	±0.0073	±9.5068
**6**	**Stem (4 + 5)**	**0.7334**	**4.4E-04**	**0.0210**	**2.8615**	**±0.0420**	**±5.7230**
7	Branches	0.2928	3.1E-04	0.0177	6.0590	±0.0355	±12.1180
8	Foliage	0.0242	6.6E-06	0.0026	10.6242	±0.0051	±21.2483
**9**	**Crown (7 + 8)**	**0.3170**	**3.6E-04**	**0.0190**	**5.9973**	**±0.0380**	**±11.9946**
**10**	**Shoot system (6 + 9)**	**1.0504**	**1.2E-03**	**0.0340**	**3.2345**	**±0.0679**	**±6.4690**
**11**	**Total tree (3 + 10)**	**1.3072**	**1.8E-03**	**0.0428**	**3.2736**	**±0.0856**	**±6.5472**

The major components and their values are indicated in bold font. SE, standard error; CI, confidence limit.

Using linear regression test, Pearson’s correlation coefficient test of significance, and distance covariance (dcov) test of independence, the BEF of taproots, lateral roots, and foliage was found to be DBH-dependent (Tables 4 and 5) (a weak dependence). Other seven component BEFs and total tree BEF were not found to have any kind of dependence on DBH (neither linear nor nonlinear). The strongest DBH-dependence was found for foliage BEF (adjusted *R* ^*2*^ = 0.2900, r = − 0.5329, dcor = 0.5874). Seven component BEFs were linearly TH-dependent (Table 6); however, using dcov test of independence, only 5 component BEFs were TH-dependent; i.e. the linear dependence of crown and shoot system BEFs on TH was not detected by dcov test of independence (Table 7). The BEF of foliage was the most strongly dependent on both DBH and TH. Component BEF values decreased with increasing TH and DBH (except for the relationship between lateral roots and DBH).

**Table 4:** **Linear regression test for dependence of biomass expansion factors** (**BEF) on diameter at breast height (DBH) in** ***A. johnsonii***

**BEF = b**_**0**_ **+ b**_**1**_ **DBH**					
**#**	**Tree component**	**b**_**0**_ **(± SE)**	**b**_**1**_ **(± SE)**	**Probability**	**Adjusted R** ^**2**^
1	Taproot + stump	0.1890 (±0.0115)	– 0.0027 (±0.0006)	0.0000	0.1768
2	Lateral roots	0.0789 (±0.0125)	0.0021 (±0.0007)	0.0017	0.0933
**3**	**Root system (1 + 2)**	**0.2679 (±0.0175)**	**– 0.0006 (±0.0009)**	**0.4963**	**– 0.0058**
4	Stem wood	0.6482 (±0.0176)	0.0005 (±0.0009)	0.5911	– 0.0078
5	Stem bark	0.0798 (±0.0075)	– 0.0002 (±0.0004)	0.6314	– 0.0084
**6**	**Stem (4 + 5)**	**0.7280 (±0.0179)**	**0.0003 (±0.0009)**	**0.7435**	**– 0.0098**
7	Branches	0.2821 (±0.0376)	0.0006 (±0.0020)	0.7583	– 0.0099
8	Foliage	0.0557 (±0.0055)	– 0.0018 (±0.0003)	0.0000	0.2870
**9**	**Crown (7 + 8)**	**0.3378 (±0.0411)**	**– 0.0012 (±0.0021)**	**0.5838**	**– 0.0076**
**10**	**Shoot system (6 + 9)**	**1.0658 (±0.0445)**	**– 0.0009 (±0.0023)**	**0.7080**	**– 0.0094**
**11**	**Total tree (3 + 10)**	**1.3336 (±0.0574)**	**– 0.0015 (±0.0030)**	**0.6192**	**– 0.0082**

The major components and their values are indicated in bold font. b_0_ and b_1_, regression parameters; SE, standard error; probability refers to the significance of the regression.

**Table 5:** **Pearson’s correlation coefficient test of significance, and distance covariance test of independence of biomass expansion factors** (**BEF) on diameter at breast height (DBH) in** ***A. johnsonii***

**BEF vs. DBH**						
		**Pearson’s correlation test**		**Distance covariance test of independence**		
**#**	**Tree component**	**r**	**Probability**	**dcov**	**dcor**	**Probability**
1	Taproot + stump	– 0.4310	1.6E-05	0.1802	0.4668	0.0150
2	Lateral roots	0.3211	0.0017	0.1670	0.4377	0.0150
**3**	**Root system (1 + 2)**	**– 0.0714**	**0.4963**	**0.1154**	**0.2546**	**0.0700**
4	Stem wood	0.0564	0.5911	0.0671	0.1430	0.7650
5	Stem bark	– 0.0504	0.6314	0.0442	0.1468	0.5200
**6**	**Stem (4 + 5)**	**0.0344**	**0.7435**	**0.0677**	**0.1428**	**0.7450**
7	Branches	0.0323	0.7583	0.1567	0.2190	0.2950
8	Foliage	– 0.5429	1.9E-08	0.1521	0.5874	0.0150
**9**	**Crown (7 + 8)**	**– 0.0575**	**0.5838**	**0.1684**	**0.2274**	**0.2450**
**10**	**Shoot system (6 + 9)**	**– 0.0394**	**0.7080**	**0.1738**	**0.2260**	**0.3300**
**11**	**Total tree (3 + 10)**	**– 0.0522**	**0.6192**	**0.2132**	**0.2432**	**0.2750**

The major components and their values are indicated in bold font. r, Perason’s correlation coefficient; dcov, distance covariance; dcor, distance correlation; probability refers to the significance of the test.

**Table 6:** **Linear regression test for dependence of biomass expansion factors** (**BEF) on total tree height (TH) in** ***Androstachys johnsonii***

**BEF = b**_**0**_ **+ b**_**1**_ **TH**					
**#**	**Tree component**	**b**_**0**_ **(± SE)**	**b**_**1**_ **(± SE)**	**Probability**	**Adjusted R** ^**2**^
1	Taproot + stump	0.2884 (±0.0248)	– 0.0120 (±0.0020)	0.0000	0.2787
2	Lateral roots	0.1178 (±0.0304)	– 0.0001 (±0.0024)	0.9585	– 0.0110
**3**	**Root system (1 + 2)**	**0.4061 (±0.0371)**	**– 0.0121 (±0.0030)**	**0.0001**	**0.1456**
4	Stem wood	0.6697 (±0.0404)	– 0.0010 (±0.0032)	0.7486	– 0.0098
5	Stem bark	0.1018 (±0.0170)	– 0.0021 (±0.0014)	0.1345	0.0137
**6**	**Stem (4 + 5)**	**0.7715 (±0.0409)**	**– 0.0031 (±0.0033)**	**0.3461**	**– 0.0011**
7	Branches	0.4745 (±0.0844)	– 0.0147 (±0.0068)	0.0314	0.0394
8	Foliage	0.1142 (±0.0118)	– 0.0073 (±0.0009)	0.0000	0.3911
**9**	**Crown (7 + 8)**	**0.5887 (±0.0900)**	**– 0.0221 (±0.0072)**	**0.0029**	**0.0835**
**10**	**Shoot system (6 + 9)**	**1.3603 (±0.0969)**	**– 0.0251 (±0.0077)**	**0.0016**	**0.0939**
**11**	**Total tree (3 + 10)**	**1.7664 (±0.1230)**	**– 0.0373 (±0.0098)**	**0.0003**	**0.1267**

The major components and their values are indicated in bold font. b_0_ and b_1_, regression parameters; SE, standard error; probability refers to the significance of the regression.

**Table 7:** **Pearson’s correlation coefficient test of significance and distance covariance test of independence of biomass expansion factors** (**BEF) on total tree height (TH) in** ***A. johnsonii***

**BEF vs. TH**						
		**Pearson’s correlation test**		**Distance covariance test of independence**		
**#**	**Tree component**	**r**	**Probability**	**dcov**	**dcor**	**Probability**
1	Taproot + stump	– 0.5353	3.2E-08	0.1106	0.5672	0.0150
2	Lateral roots	– 0.0055	0.9585	0.0516	0.2679	0.1550
**3**	**Root system (1 + 2)**	**– 0.3936**	**9.5E-05**	**0.0918**	**0.4009**	**0.0150**
4	Stem wood	– 0.0337	0.7486	0.0370	0.1562	0.6350
5	Stem bark	– 0.1564	0.1345	0.0249	0.1636	0.5250
**6**	**Stem (4 + 5)**	**– 0.0988**	**0.3461**	**0.0439**	**0.1833**	**0.3750**
7	Branches	– 0.2233	0.0314	0.0844	0.2334	0.2400
8	Foliage	– 0.6306	1.2E-11	0.0835	0.6386	0.0150
**9**	**Crown (7 + 8)**	**– 0.3057**	**0.0029**	**0.1089**	**0.2910**	**0.0600**
**10**	**Shoot system (6 + 9)**	**– 0.3220**	**0.0016**	**0.1131**	**0.2911**	**0.1300**
**11**	**Total tree (3 + 10)**	**– 0.3691**	**0.0003**	**0.1495**	**0.3376**	**0.0400**

The major components and their values are indicated in bold font. r, Perason’s correlation coefficient; dcov, distance covariance; dcor, distance correlation; probability refers to the significance of the test.

### Biomass density

Total tree biomass was approximately 25% higher than AGB (Table 8). The root system, stem, and crown observed biomass densities of 29.62, 84.57, and 36.55 Mg ha^−1^, respectively. Stem biomass density accounted for approximately 70% of AGB and 56% of the total tree biomass density. As expected, the estimates of biomass densities are as precise as the estimates of BEFs.

**Table 8:** **Biomass density (** ***W***_***h***_ **), variance (VAR**_**Wh**_ **), standard error (SE), and 95% confidence intervals (CI) for each component in** ***Androstachys johnsonii*** **trees**

**#**	**Tree component**	**W**_**h**_ **(Mg ha** ^**–1**^ **)**	**VAR**_**Wh**_ **(Mg** ^**2**^ **ha** ^**–2**^ **)**	**SE (Mg ha** ^**–1**^ **)**	**SE (%)**	**95% CI (Mg ha** ^**–1**^ **)**	**95% CI (%)**
1	Taproot + stump	16.2192	0.4725	0.6874	4.2382	±1.3748	±8.4764
2	Lateral roots	13.4005	0.5882	0.7669	5.7232	±1.5339	±11.4465
**3**	**Root sytem (1 + 2)**	**29.6197**	**1.3296**	**1.1531**	**3.8930**	**±2.3062**	**±7.7860**
4	Stem wood	75.7526	4.8413	2.2003	2.9046	±4.4006	±5.8092
5	Stem bark	8.8182	0.1757	0.4192	4.7534	±0.8383	±9.5068
**6**	**Stem (4 + 5)**	**84.5708**	**5.8565**	**2.4200**	**2.8615**	**±4.8400**	**±5.7230**
7	Branches	33.7612	4.1845	2.0456	6.0590	±4.0912	±12.1180
8	Foliage	2.7923	0.0880	0.2967	10.6242	±0.5933	±21.2483
**9**	**Crown (7 + 8)**	**36.5535**	**4.8058**	**2.1922**	**5.9973**	**±4.3844**	**±11.9946**
**10**	**Shoot system (6 + 9)**	**121.1243**	**15.3491**	**3.9178**	**3.2345**	**±7.8356**	**±6.4690**
**11**	**Total tree (3 + 10)**	**150.7440**	**24.3521**	**4.9348**	**3.2736**	**±9.8696**	**±6.5472**

The major components and their values are indicated in bold font. SE, standard error; CI, confidence limit.

### Root-to-shoot ratio

The average root-to-shoot ratio was 0.24 (minimum = 0.07, maximum = 0.35, SD = 0.04, CV = 16.8%). The uncertainty (SE) of the estimated R/S was 3.4% (CI = 6.78%). The root-to-shoot ratio was neither linear nor nonlinearly dependent on any of the four variables (DBH, TH, AGB, and BGB) (Tables 9 and 10). The BGB density calculated based on R/S was 29.26 Mg ha^−1^ (SE = 3.4%), which was 1.20% smaller and 13.73% more precise than the BGB density estimate based on BEF.

**Table 9:** **Linear regression test of dependence of root-to-shoot ratio on diameter at breast height (DBH), total height (TH), aboveground biomass (AGB), belowground biomass (BGB), and total biomass (TB) in** ***Androstachys johnsonii*** **trees**

**#**	**Regression equation**	**b**_**0**_ **(± SE)**	**b**_**1**_ **(± SE)**	**Probability**	**Adjusted R** ^**2**^
1	R/S = b0 + b1DBH	0.24051 (±0.01080)	0.00006 (±0.00057)	0.9154	– 0.0109
2	R/S = b0 + b1H	0.27807 (±0.02454)	– 0.00296 (±0.00196)	0.1347	0.0137
3	R/S = b0 + b1AGB	0.27015 (±0.00662)	0.00001 (±0.00003)	0.7812	– 0.0101
4	R/S = b0 + b1BGB	0.23354 (±0.00634)	0.00017 (±0.00010)	0.0997	0.0188
5	R/S = b0 + b1TB	0.23876 (±0.00659)	0.00001(±0.00002)	0.5802	– 0.0076

b_0_ and b_1_, regression parameters; SE, standard error; probability refers to the significance of the regression.

**Table 10:** **Pearson’s correlation coefficient test of significance and distance covariance test of independence of root-to-shoot ratio on diameter at breast height (DBH), total height (TH), aboveground biomass (AGB), belowground biomass (BGB), and total biomass (TB) in** ***Androstachys johnsonii*** **trees**

**#**	**Pair of variables**	**Pearson’s correlation test**		**Distance covariance test of independence**		
		**r**	**Probability**	**dcov**	**dcor**	**Probability**
1	R/S vs. DBH	0.0112	0.9154	0.0554	0.1573	0.8650
2	R/S vs. H	– 0.1563	0.1347	0.0473	0.2662	0.0650
3	R/S vs. AGB	0.0292	0.7812	0.2475	0.1518	0.8200
4	R/S vs. BGB	0.1718	0.0997	0.1575	0.1951	0.2900
5	R/S vs. TB	0.0581	0.5802	0.2763	0.1519	0.8100

r, Perason’s correlation coefficient; dcov, distance covariance; dcor, distance correlation; probability refers to the significance of the test.

## Discussion

### Component biomass expansion factors and biomass density

A wider range of DBH was measured during the first sampling phase than during the second phase. However, the DBH of *A. johnsonii* rarely exceeds 35 cm (here, <1% of trees during the first sampling phase). Although large variation in TH was observed during both phases, <4% of the trees had TH >16 m or <5 m, which indicated that phase-2 samples were representative of the phase-1 samples, and thus the values could be extrapolated.

BEF values are generally calculated from the ratio of tree component or total tree biomass (*W*_*h*_) to stem or merchantable timber volume (*v*) [4,6,9-12] or biomass (*W*_*s*_) [7,8,13-15]. We calculated BEFs using the first option (here called BEF_1_) with total stem volume. The stem BEF value was 0.7334 Mg m^−3^, which meant that stem biomass (in Mg) was 0.7334-fold larger than stem volume (in m^3^). Therefore, BEF computed according to biomass (the second option, here called BEF_2_) can be calculated as a function of BEF_1_ as $BE{F}_2=\frac{W_h}{0.7334\times v}=\frac{1}{0.7334}\times BE{F}_1$.

Since BEF_2_ is obtained by multiplying BEF_1_ by a constant, the relationship between BEF_2_ and tree size (DBH and TH) is the same as that between BEF_1_ and tree size (both relationships will be either significant or insignificant). Therefore, trends in BEF values calculated here using the first option were compared indiscriminately to those calculated using either option.

The same principle holds for BEF values calculated using merchantable timber volume or biomass; because merchantable timber volume or biomass are obtained by multiplying stem biomass or volume by the merchantable fraction of the total stem (ratio of timber volume to stem volume) [8], which is a constant. For most trees, this fraction is very close to 1 [8], which makes BEF values calculated with merchantable volume or biomass very close to those calculated with stem volume or biomass.

We preferred the use of BEF_1_ to BEF_2_ because stem volume is easily measured destructively than stem biomass, and volume is the main variable of interest in most forest inventories. In addition, stem volume was preferred to merchantable volume because merchantable height is sensitive to personal judgment and thus is more subjective than stem height, especially for standing trees. Merchantable tree height measurement (e.g. to 7 cm top diameter as defined by Lehtonen et al. [4], Lehtonen et al. [9], Edwards and Christie [16], and Black et al. [17]) in standing trees is subjective and more susceptible to measurement error than total tree height, because the 7 cm top diameter on the stem is difficult to identify than the tip of the tree. Moreover, in most tropical tree species, and especially in broadleaf species (as opposed to coniferous), taking a minimum top diameter of 7 cm to define merchantable tree height is somewhat impractical because the merchantable height is limited by branching, irregular form or defects which causes inconsistence in the top diameter definition.

Because stem volume is the auxiliary variable for all tree components, estimation of biomass density based on BEF achieves the property of additivity automatically for the major components (root system, shoot system, stem, and crown) and for total tree biomass, without additional efforts, which is a great advantage.

The BEF values estimated here fall in the range of many estimates obtained worldwide e.g. [4-7,10,14,18], especially with those of whole-tree BEF. For example, Kamelarczyk [18] reported whole-tree BEF values from 0.06 to 2.90 for 17 miombo tree species in Zambia. Estimates of aboveground and total tree BEF compiled for Africa by the FAO [19] were 1.5 and 1.9, 43% and 45% larger than our estimates, respectively; FAO values of eastern Africa were 2.3 for aboveground BEF and 2.9 for total BEF, which were more than two-fold higher than our estimates. However, the FAO’s global-scale estimates (1.0 for aboveground BEF and 1.3 for total tree BEF) were closer to our findings [19].

Reports on the dependence of BEF values on DBH and TH vary, from strong reverse dependence [3-7] to weak reverse dependence or independence [10]. Here, we found component BEFs to be either independent or have a weak reverse dependence on DBH and TH, which indicated that small and large *A. johnsonii* trees contain approximately the same quantity of biomass per unit volume.

Ducta et al. [6] maintained that the reverse dependence of BEF on tree size is a result of an inverse relationship between wood density and tree size. We did not observe variation in stem wood and stem bark densities according to DBH and TH for *A. johnsonii* (adj. *R* ^2^ < 0.0309, *P* >0.05), and a very weak relationship was found between total stem density and DBH (adj. *R* ^2^ = 0.1342, *P* =0.0002) and TH (adj. *R* ^2^ = 0.0661, *P* =0.0072). These results explained the independence or weak dependence of component BEF values on tree size.

Our observation of a slightly stronger relationship between BEF values and TH compared to DBH was consistent with the findings of other researchers [4,6,8,12], but contradicted the report by Sanquetta et al. [7].

The dependence of component BEFs (taproots, lateral roots, and foliage) on DBH detected by the linear regression test and Pearson’s correlation coefficient test of significance were also detected by the dcov test of independence; suggesting that, the most pronounced dependence of these component BEFs on DBH is linear, since dcov test measures all types of dependence (linear and nonlinear). On the other hand, the absence of dependence of other 7 components and total tree BEFs on DBH by either method, suggests that there is not any type of dependence (linear, nonlinear or nonmonotone) of those component BEFs on DBH.

A linear dependence of crown and shoot system BEFs on TH was detected by the linear regression test and Pearson’s correlation coefficient test of significance. However, this dependence was not detected by the dcov test of independence, which may suggest that this linear dependence is casual.

The finding of independence or weak dependence of the BEF on tree size might be related to the minimum DBH measured in the phase-2 (DBH ≥ 5 cm). It has been reported by Brown et al. [3], Saquentta et al. [7], Marková and Pokorný [10], and Soares and Tomé [20] that the decrease of the BEF with tree size reaches an asymptote at a given tree size. This is presumably due to stabilization of growth rate [7].

The finding of independence or weak dependence of the BEF on tree size suggests that, for *A. johnsonii*, constant component BEF values can be accurately used within the interval of harvested tree sizes (5 ≤ DBH ≤ 32, Table 1), in contrast to findings by Brown et al. [3] and Sanquetta et al. [7]. Here, further research would be needed to reveal the relationship between tree component BEFs and tree’s DBH ≤ 5 cm.

We defined the stem as the length from the top of the stump to the height corresponding to 2.5 cm diameter. Differences among stem definitions (e.g. different stump height or different minimum top diameter, stump considered as part of the stem) would affect the BEF estimates.

It was difficult to compare our 4 major and 6 minor component BEF and biomass density values, because few similar studies have been performed in African and Mozambican woodlands. The majority of available studies provide estimates of BEF and biomass for shoot systems and occasionally for the whole tree. Our estimated AGB density (121 Mg ha^−1^) was within the range reported by Lewis et al. [21] for tropical African forests (114–749 Mg ha^−1^) and by Brown [11] for hardwood forests (75–175 Mg ha^−1^); and lower than estimates for closed tropical forests (144–513 Mg ha^−1^) [22,23]. Our AGB density estimate was higher than Brown and Lugo’s estimate for open tropical forests (50 Mg ha^−1^) [23].

Estimates of stem-wood biomass density by Brown and Lugo [23] for undisturbed, logged, and unproductive tropical African forests were 148.6, 41.2, and 36 Mg ha^−1^, respectively, while our estimate was 75.75 Mg ha^−1^. Our estimated whole-tree biomass density (approximately 150 Mg ha^−1^) was similar to those for unproductive (129 Mg ha^−1^) and logged (179 Mg ha^−1^) tropical African forests, and smaller than Brown and Lugo’s estimate for undisturbed forests (238 Mg ha^−1^) [23]. However, the estimates by Brown and Lugo [22,23] were performed more than 4 decades ago, and thus, they might not reflect the current situation.

Our estimated AGB density (121 Mg ha^−1^) are in agreement with those estimated for Mozambique by Brown [24] for dense forests in moist-dry season (120 Mg ha^−1^) and in moist-short dry season (130 Mg ha^−1^) but are higher compared to dense forests in dry season (70 Mg ha^−1^). Yet, mecrusse woodlands (*A. johnsonii* stands) are typically from dry season [25-29], implying that the biomass productivity of mecrusse woodlands is, approximately, twice as larger than the average productivity of dense forests in dry season in Mozambique.

The estimated uncertainty in our BEF values (2.9%–10.6%) was lower than that of Lehtonen et al. (3%–21%) [4,9] and Jalkanen et al. (4%–13%) [30]. The component biomass and stem volume values used here to calculate BEF were obtained directly using destructive sampling, whereas Lehtonen et al. [4,9] and Jalkanen et al. [30] were based on values obtained indirectly using regression models. These different approaches might explain the differences among BEF estimates and the higher uncertainty reported by those authors, because they also incorporate uncertainty from the regression models.

The default IPCC aboveground BEF for tropical broadleaf species is 1.5 ± 0.2 (SE = 6.67%) [31]. This BEF value is 43% larger and 200% more uncertain than our estimated aboveground BEF (1.05 ± 0.07 Mg m^−3^, SE = 3.23%). The default IPCC BEF-based AGB density was 173 ± 28 Mg ha^−1^ (SE = 8.10%), 43% larger and 107% more uncertain than our estimated AGB density (121 ± 6.47 Mg ha^−1^, SE = 3.23%). The default IPCC BEF-based AGB density is not in agreement with the estimated AGB density for Mozambique by Brown [24].

### Root-to-shoot ratios

The average root-to-shoot ratio found in this study (0.24 or 1:4) was larger than that observed by some authors, such as 1:5 (0.2) reported by Kramer [32], 1:6 (0.17) reported by Perry [33], and 0.17 reported by Sanquetta et al. [7]. The findings of these authors suggest that AGB is 5- to 6-fold greater than BGB, but our finding that AGB is, on average, almost 4-fold higher than BGB was consistent with the default IPCC root-to-shoot ratio of 1:4 (0.25) [31]. We determined BGB by complete removal of the root system, including the root collar and fine roots. Estimates of R/S may vary greatly if the root system is partially removed, as performed by many authors e.g. [7,8,34-37], if the depths of excavation are predefined [7,37,38], if fine roots are excluded [39-41]. R/S values may also vary if root sampling procedures are applied, for example, where only a number of roots from each root system is fully excavated, and then the information from the excavated roots is used to estimate biomass for the roots not excavated [42-44]. Different estimates of R/S can also be obtained if the stump is considered as part of the stem, as in Segura and Kanninen [14].

Wang et al. [45] similarly observed little variation in the relationship between R/S and tree diameter. However, different results were obtained by Mokany et al. [2] for root-to-shoot ratios in different terrestrial biomes (forests, woodland, shrublands and grasslands), where the ratios decreased significantly with increasing shoot biomass, tree height, and DBH. Our findings were also inconsistent with those of Sanquetta et al. [7], who found that R/S decreased as DBH and TH increased. This might be presumably because *A. johnsonii*, as a tropical native species, has a very low and/or constant growth rate within the interval of harvested DBH, as opposed to the planted *Pinus spp.* studied by Sanqueta et al. [7].

As in the case of the BEF, the finding of independence of R/S on tree size might be related to the minimum DBH measured in the phase-2 (DBH ≥ 5 cm). Mokany et al. [2], Saquentta et al. [7], Jenkins et al. [46], and Zhou and Hemstrom [47] have shown that decrease of R/S with tree size reaches an asymptote at a given tree size, presumably due to stabilization of growth rate [7]. Inclusion of trees with DBH ≤ 5 cm could cause variation of R/S with tree size. Therefore, researches are also needed here to reveal the relationship between R/S and tree’s DBH ≤ 5 cm.

## Conclusions

The belowground, aboveground, and whole-tree BEFs were 0.26 ± 0.02 (SE = 3.89%), 1.05 ± 0.07 (SE = 3.23%), and 1.30 ± 0.09 Mg m^−3^ (SE = 3.27%), respectively; equivalent to, approximately, the following BEF-based biomass densities: 30 ± 2.31, 121 ± 7.84, and 151 ± 9.87 Mg ha^−1^, respectively.

We observed that component BEFs in *Androstachys johnsonii* Prain were independent or only weakly dependent on tree size (DBH and TH), and that TH was more important that DBH in explaining BEF. Therefore, we suggested that constant component BEF values can be accurately used within the interval of harvested tree sizes. The root-to-shoot ratio (average = 0.24 ± 0.02; SE = 3.4%) was not dependent on tree height, DBH, AGB, or BGB.

## Methods

### Study area

**Figure 2:**
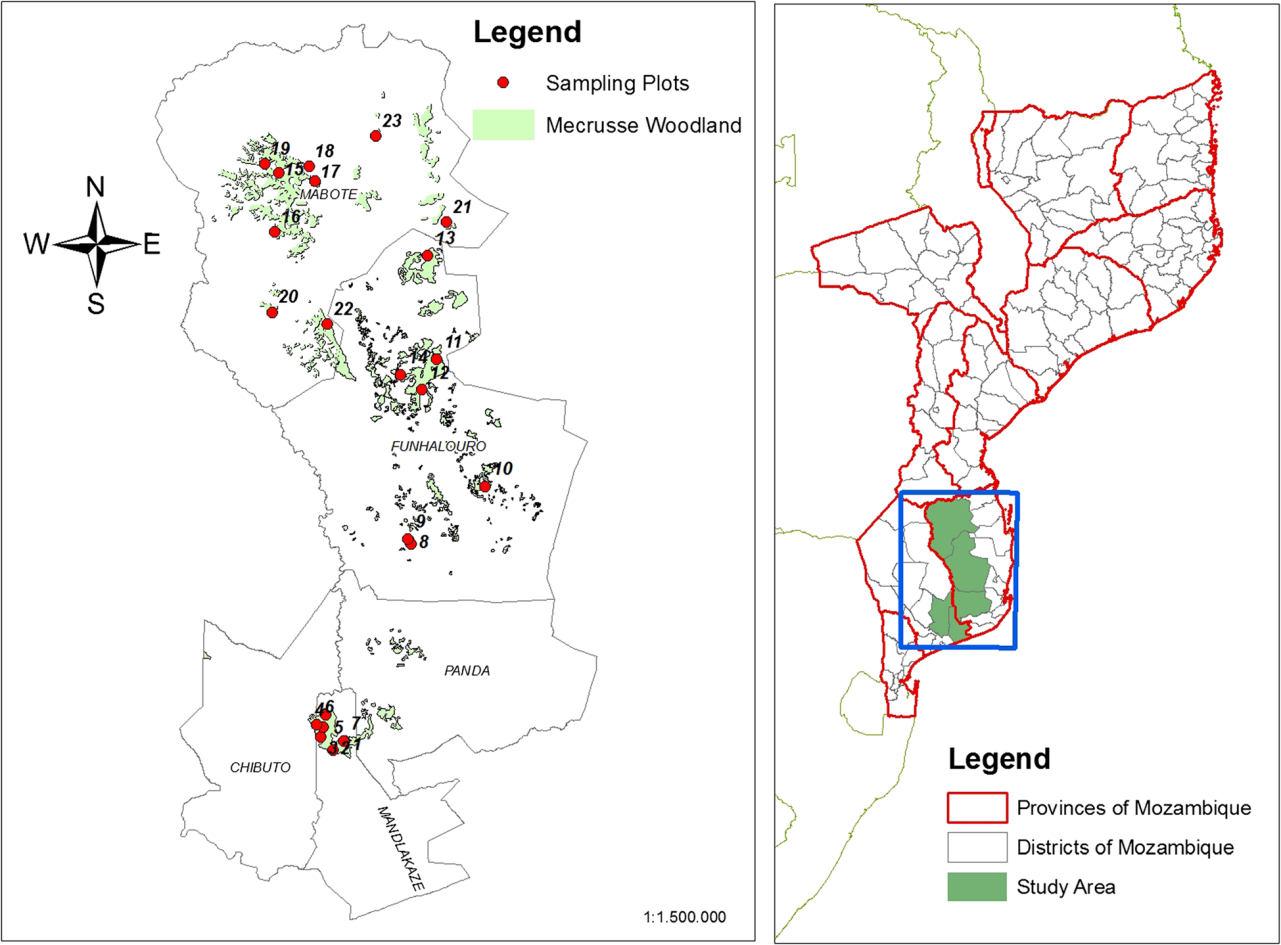
Distribution of sampling plots in mecrusse forest patches.

Mecrusse is a forest type in which the dominant canopy species is *Androstachys johnsonii*, the relative cover of which varies from 80% to 100% [48]. In Mozambique (18°15′S, 35°00′E), mecrusse woodlands are mainly found in Inhambane and Gaza provinces in Massangena, Chicualacuala, Mabalane, Chigubo, Guijá, Mabote, Funhalouro, Panda, Mandlakaze, and Chibuto districts. The easternmost mecrusse forest patches, located in Mabote, Funhalouro, Panda, Mandlakaze, and Chibuto districts, were defined as the study area (Figure 2) and encompassed 4,502,828 ha [49], of which 226,013 ha (5%) were mecrusse woodlands. The climate throughout the study area is dry tropical, with the exception of humid tropical areas in western Panda and southwestern Mandlakaze districts [25-29,49]; a warm or rainy season occurs from October to March, and a cool or dry season occurs from March to September [25-29].

The mean annual temperature generally exceeds 24°C, and mean annual precipitation varies from 400 to 950 mm [25-29,49]. According to the United States Food and Agriculture Organization (FAO) classification [50], soils are mainly Ferralic Arenosols across more than 70% of the study area [49]. Arenosols, Umbric Fluvisols, and Stagnic soils are predominant in the northernmost part of the study area [49]. There is a shortage of water resources and precipitation throughout the study area; only Chibuto and Mandlakaze districts have water resources [25-29,49].

### Data collection

We used a two-phase sampling design to determine stem volume and biomass. In the first phase, we measured diameter at breast height (DBH) and stem height of 3574 trees (*m*_1_) in 23 randomly located circular plots (20-m radius) (Figure 2) for estimation of stem volume; only trees with DBH ≥ 5 cm were considered. In the second phase, 93 trees (*m*_2_) (DBH ≥ 5 cm) were randomly selected from those analysed during the first phase for destructive measurement of biomass and stem volume. The felled trees were divided into the following components: (1) taproot; (2) lateral roots; (3) root system (1 + 2); (4) stem wood; (5) stem bark; (6) stem (4 + 5); (7) branches; (8) foliage; (9) crown (6 + 7); (10) shoot system (6 + 9); and (11) whole tree (3 + 10). Tree components were sampled and the dry weights estimated as follows.

### Root system

The stump height was predefined as being 20 cm from the ground level for all trees and considered as part of the taproot, as recommended by Parresol [51] and because in larger *A. johnsonii* trees this height (20 cm) is affected by root buttress; therefore, the root collar was also considered part of the taproot. The root system was divided into 3 sub-components: fine lateral roots, coarse lateral roots, and taproot. Lateral roots with diameters at insertion point on the taproot < 5 cm were considered as fine roots and those with diameters ≥ 5 cm were considered as coarse roots.

**Figure 3:**
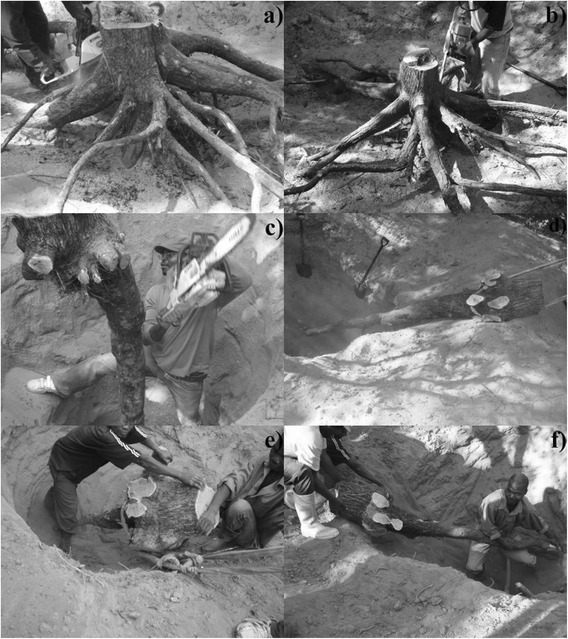
Separation of lateral roots from the root collar/taproot **(a, b, c)**, and removal of the taproot including the root collar and the stump **(d, e, f)**.

First, the root system was partially excavated to the first node, using hoes, shovels, and picks; to expose the primary lateral roots (Figure 3a, b). The primary lateral roots were numbered and separated from the taproot with a chainsaw (Figure 3a, b) and removed from the soil, one by one. This procedure was repeated in the subsequent nodes until all primary roots were removed from the taproot and the soil. Finally, the taproot was excavated and removed (Figure 3 c–f). The complete removal of the root system was relatively easy because 90% of the lateral roots of *A. johnsonii* are located in the first node, which is located close to ground level (Figure 3 a–c); the lateral roots grow horizontally to the ground level, do not grow downwards; and because the taproots had, at most, only 4 nodes and at least 1 node (at ground level). The root system was removed completely, so the depth of excavation depended on the depth of the taproot.

Fresh weight was obtained for the taproot, each coarse lateral root and for all fine lateral roots. A sample was taken from each sub-component, fresh weighed, marked, packed in a bag, and taken to the laboratory for oven drying. For the taproot, the samples were two discs, one taken immediately below the ground level and another from the middle of the taproot. For the coarse lateral roots, two discs were also taken, one from the insertion point on the taproot and another from the middle of it. For fine roots the sample was 5 to 10% of the fresh weight of all fine lateral roots. Oven drying of all samples was done at 105°C to constant weight, hereafter, referred to as dry weight.

### Stem wood and stem bark

Felled trees were scaled up to a 2.5 cm top diameter. The stem was defined as the length of the trunk from the stump to the height that corresponded to 2.5 cm diameter, to standardize with the definitions of fine branches. The remainder (from the height corresponding to 2.5 cm diameter to the tip of the tree) was considered a fine branch.

First, we divided the stem of each felled tree into 10 segments of equal length, and we measured the diameter of each segment at the midpoint, starting from the bottom of the stem, for volume and form factor determination using Hohenadl formula. The stem was, then, divided into sections, the first with 1.1 m length, the second with 1.7 m, and the remaining with 3 m, except the last, the remainder, which length depended on the length of the stem.

Discs were removed at the bottom and top of the first section, and on the top of the remaining sections; i.e.: discs were removed at heights of 0.2 m (stump height), 1.3 m (breast height), 3 m, and the successive discs were removed at intervals of 3 m to the top of the stem, and their fresh weights measured using a digital scale.

Diameters over and under bark were taken from the discs in the North–south direction (previously marked on the standing tree) with the help of a ruler. The volumes over and under the bark of the stem were obtained by summing up the volumes of each section calculated using Smalian’s formula [52]. Bark volume was obtained from the difference between volume over bark and volume under bark.

The discs were dipped in drums filled with water, until constant weight (3 to 4 months), for its saturation and subsequent determination of the saturated volume and basic density. The saturated volume of the discs was obtained based on the water displacement method [53] using Archimedes’ principle. This procedure was done twice: before and after debarking; hence, we obtained saturated volume under and over the bark.

Wood discs and respective barks were oven dried at 105°C to constant weight. Basic density was obtained by dividing the oven dry weight of the discs (with and without bark) by the relevant saturated wood volume [54,55]. Therefore, two distinct basic densities were calculated: (1) basic density of the discs with bark and (2) basic density of the discs without bark.

We estimated the basic density at point of geometric centroid of each section using the regression function of density over height [56]. This density value was taken as representative of each section [56].

### Crown

The crown was divided into two sub-components: branches and foliage. Primary branches, originating from the stem, were classified in two categories: primary branches with diameters at the insertion point on the stem ≥ 2.5 cm were classified as large branches, and those with diameter < 2.5 cm were classified as fine branches. Large branches were sampled similarly to coarse roots, and fine branches and foliage were sampled similarly to fine roots.

### Tree component dry weights

We determined dry weight of the taproot, lateral roots, branches, and foliage by multiplying the ratio of fresh- to oven-dry weight of each sample by the total fresh weight of the relevant component. Dry weights of the root system and crown were obtained by summing up the relevant sub-components’ dry weights. Dry weights of each stem section (with and without bark) were obtained by multiplying respective densities by relevant stem section volumes.

Stem (wood + bark) and stem wood dry weights were obtained by summing up each section’s dry weight with and without bark, respectively. The dry weight of stem bark was determined from the difference between the dry weights of the stem and stem wood. We determined the dry weight of major components (root system, shoot system, and crown) and the whole tree by summing the dry weights of their constituent components.

### Data processing and analysis

Stem volume was computed using Hohenadl’s method (Eq. 1) [57].

1 $$ {v}_{i2}=\frac{\varPi L}{40}\left({d}_{.05}^2+{d}_{.15}^2+{d}_{.25}^2+{d}_{.35}^2+{d}_{.45}^2+{d}_{.55}^2+{d}_{.65}^2+{d}_{.75}^2+{d}_{.85}^2+{d}_{.95}^2\right)\left[{\mathrm{m}}^3\right] $$

where *v*_*i2*_ is the stem volume of the *i* ^*th*^ tree from the second sampling phase, *L* is the stem length (in meters), and *d*_*.i*_ is the diameter (in meters) measured at the proportional distance along the stem of the *i* ^*th*^ tree.

The individual stem volume of the *i* ^*th*^ tree of the *j* ^*th*^ plot from the first sampling phase (*v*_*ij*1_) was calculated using Eq. 2 as follows:

2 $$ {v}_{ij1}=\frac{\varPi }{4}DB{H}^2\times H\times {f}_h\kern0.5em \left[{\mathrm{m}}^3\right] $$

where *H* is stem height and *f*_*h*_ is the Hohenadl form factor of the trees from the second sampling phase, obtained using Eq. 3 as:

3 $$ {f}_h=0.1\left(1+\frac{d_{.15}^2}{d_{.05}^2}+\frac{d_{.25}^2}{d_{.05}^2}+\frac{d_{.35}^2}{d_{.05}^2}+\frac{d_{.45}^2}{d_{.05}^2}+\frac{d_{.55}^2}{d_{.05}^2}+\frac{d_{.65}^2}{d_{.05}^2}+\frac{d_{.75}^2}{d_{.05}^2}+\frac{d_{.85}^2}{d_{.05}^2}+\frac{d_{.95}^2}{d_{.05}^2}\right)\left[\mathrm{dimensionless}\right] $$

The main auxiliary variable (the first-phase variable) is the stand-level stem volume (m^3^ ha^−1^), estimated from Eq. 4 as follows:

4 $$ {V}_1=\frac{{\displaystyle \sum_{j=1}^n{\displaystyle \sum_{i=1}^{m_j}{v}_{ij1}}}}{n\times a}={\overline{v}}_1\times {N}_1\left[{\mathrm{m}}^3{\mathrm{ha}}^{-1}\right] $$

where *m*_*j*_ is the number of trees in the *j* ^*th*^ plot, *n* is the number of plots, *a* is the plot area (ha), ${\overline{v}}_1$ is the average stem volume of the trees of the first phase (m^3^), and *N*_1_ is the average number of trees per hectare estimated from the first sampling phase. Stem height of trees from the first phase was obtained by subtracting predefined stump height from the whole-tree height (TH) to standardize the definitions of stem height and stem length (for phase-1 trees).

The component biomass expansion factors for each tree (*BEF*_*hi*_) in the second sampling phase were calculated as the ratio of tree component biomass *w*_*hi2*_ to stem volume *v*_*i2*_ [4-6,9,10] (Eq. 5) and the average was taken as the component BEF (Eq. 6) of the woodland. This process enabled us to convert stem volume to biomass. The root-to-shoot ratio (R/S) was determined as the ratio of BGB to AGB [1,2,40] for each tree (Eq.7); the average value was taken as the overall vegetation R/S (Eq. 8).

5 $$ BE{F}_{hi}=\frac{w_{hi2}}{v_{i2}}\kern0.5em \left[\mathrm{Mg}\kern0.5em {\mathrm{m}}^{-3}\right] $$

6 $$ BE{F}_h=\frac{{\displaystyle \sum_{i=1}^{m_2} BE{F}_{hi}}}{m_2}\kern0.62em \left[\mathrm{Mg}{\mathrm{m}}^{-3}\right] $$

7 $$ {\left(R/S\right)}_i=\frac{w_{Roo{t}_{i2}}}{w_{Shoo{t}_{i2}}}\left[\mathrm{dimensionless}\right] $$

8 $$ R/S=\frac{{\displaystyle \sum_{i=1}^{m_2}{\left(R/S\right)}_i}}{m_2}\left[\mathrm{dimensionless}\right] $$

where *BEF*_*hi*_ is the BEF of the *h* ^*th*^ component of the *i* ^*th*^ tree; *w*_*hi2*_ is biomass of the *h* ^*th*^ component of the *i* ^*th*^ tree measured during the second phase; *BEF*_*h*_ is the average BEF of the *h* ^*th*^ component; *R/S*_*i*_ is the root-to-shoot ratio of the *i* ^*th*^ tree; ${w}_{{}_{Roo{t}_{{}_{i2}}}}$ and ${w}_{Shoo{t}_{i2}}$ represent BGB and AGB, respectively, of the *i* ^*th*^ tree of the second phase; and *m*_2_ is the total number of trees in the second sampling phase.

The average tree component biomass density *W*_*h*_ (Mg ha^−1^) was estimated as the product of the respective component *BEF*_*h*_ values and *V*_*1*_ (Eq. 9):

9 $$ {W}_h= BE{F}_h\times {V}_1={W}_{hi}\times {N}_1\left[\mathrm{Mg}\kern0.5em {\mathrm{ha}}^{\hbox{--} 1}\right] $$

where

10 $$ {W}_{hi}= BE{F}_h\times {\overline{v}}_1\left[\mathrm{Mg}\right] $$

is the estimated average component biomass per tree, which yields *W*_*h*_ when multiplied by the number of trees per hectare.

*BEF*_*hi*_ (Eq. 5) is the ratio of biomass of a tree component to stem volume; therefore, *BEF*_*h*_ (Eq. 6) is a mean ratio (not a ratio of means). These variables represent double sampling with mean-of-ratios estimators and dependent phases, and the uncertainty (variance and standard error) of the estimated *BEF*_*h*_ and *W*_*h*_ must be computed accordingly (as for R/S).

We calculated the variance of the estimated *W*_*hi*_ (Eq. 10) according to Freese [58,59]:

11 $$ VA{R}_{W_{hi}}={\overline{v}}_1^2\left(\frac{S_{BE{F}_h}^2}{m_2}\right)\left(1-\frac{m_2}{m_1}\right)+\frac{S_{w_{h2}}^2}{m_1}\left(1-\frac{m_1}{M}\right)\left[{\mathrm{Mg}}^2\right] $$

Rearranging Eq. 10 as $BE{F}_h=\frac{W_{hi}}{{\overline{v}}_1},$ the variance of the estimated *BEF*_*h*_ becomes [60]:

12 $$ VA{R}_{BE{F}_h}=\frac{VA{R}_{W_{hi}}}{{\overline{v}}_1^2}=\left(\frac{S_{BE{F}_h}^2}{m_2}\right)\left(1-\frac{m_2}{m_1}\right)+\left(\frac{1}{{\overline{v}}_1^2}\right)\left(\frac{S_{w_{h2}}^2}{m_1}\right)\left(1-\frac{m_1}{M}\right)\left[{\mathrm{Mg}}^2{\mathrm{m}}^{\hbox{--} 6}\right] $$

Similarly, the variance of the estimated *W*_*h*_ is [60]:

13 $$ VA{R}_{W_h}={N}_1^2\times \left[{\overline{v}}_1^2\left(\frac{S_{BE{F}_h}^2}{m_2}\right)\left(1-\frac{m_2}{m_1}\right)+\frac{S_{w_{h2}}^2}{m_1}\left(1-\frac{m_1}{M}\right)\right]\left[{\mathrm{Mg}}^2{\mathrm{ha}}^{\hbox{--} 2}\right] $$

and the variance of the estimated R/S is

14 $$ VA{R}_{R/S}=\left(\frac{S_{R/S}^2}{m_2}\right)\left(1-\frac{m_2}{m_1}\right)+\left(\frac{1}{{\overline{w}}_{Shoo{t}_1}^2}\right)\left(\frac{S_{w_{Root2}}^2}{m_1}\right)\left(1-\frac{m_1}{M}\right)\left[\mathrm{dimensionless}\right] $$

where

15 $$ {S}_{BE{F}_h}^2=\frac{{\displaystyle \sum BE{F}_{hi}^2-\frac{{\left({\displaystyle \sum BE{F}_{hi}}\right)}^2}{m_2}}}{m_2-1}\left[{\mathrm{Mg}}^2{\mathrm{m}}^{-6}\right] $$

is the variance of *BEF*_*h*_ for the second phase;

16 $$ {S}_{w_{h2}}^2=\frac{{\displaystyle \sum {w}_{hi2}^2-\frac{{\left({\displaystyle \sum {w}_{hi2}}\right)}^2}{m_2}}}{m_2-1}\left[{\mathrm{Mg}}^2\right] $$

is the variance of *w*_*h2*_; *w*_*h2*_ is the component biomass for the second phase;

17 $$ {S}_R^2=\frac{{\displaystyle \sum {\left(R/S\right)}_i^2-\frac{{\left({\displaystyle \sum {\left(R/S\right)}_i}\right)}^2}{m_2}}}{m_2-1}\left[\mathrm{dimensionless}\right] $$

is the variance of R/S for the second phase;

18 $$ {S}_{w_{Root2}}^2=\frac{{\displaystyle \sum {w}_{Roo{t}_{i2}}^2-\frac{{\left({\displaystyle \sum {w}_{Roo{t}_{i2}}}\right)}^2}{m_2}}}{m_2-1}\left[{\mathrm{Mg}}^2\right] $$

is the variance of ${w}_{Roo{t}_2}$; ${w}_{Roo{t}_2}$ is the BGB of trees of the second phase; ${\overline{w}}_{Shoo{t}_1}$ is the average AGB per tree for the first sampling phase; and *m*_*1*_, *m*_*2*_, and *M* are the number of trees in the first sampling phase, the second sampling phase, and the entire population, respectively. The finite population correction factor $\left(1-\frac{m_1}{M}\right)$ was eliminated because *m*_*1*_ was very small relative to *M*, which was unknown.

The square root of Eqs. 12 and 13 is the absolute standard error of the estimated *BEF*_*h*_ and *W*_*h*_, respectively; dividing these values by *BEF*_*h*_ and *W*_*h*_ and then multiplying them by 100 provides the respective percent standard error. The absolute and percent 95% confidence limits (CI) are computed by multiplying the absolute and percent standard error by the Student’s *t*-value (*t*). The absolute and percent standard errors of R/S are computed analogously.

The percent 95% confidence limit (Eq. 19) is also referred as percent sampling error [61].

19 $$ 95\%C{I}_{\%}={E}_{\%}=\pm \frac{t\times SE}{\overline{X}}\times 100\kern0.5em \left[\%\right] $$

where *SE* is the standard error and $\overline{X}$ is the average *BEF*_*h*_ *, W*_*h*_ or R/S.

In this study, uncertainty is expressed as the percent SE and as the percent 95% CI to facilitate comparison with existing studies as, for our knowledge, the existing studies reporting BEFs and R/S with known uncertainty use either percent SE [4,9,30] or percent 95% CI [31,39,62] to express the uncertainty.

The dependence of the component BEF values on DBH and TH was analysed by linear regression of *BEF*_*h*_ against DBH and TH and testing the significance of the regression against the null hypothesis of slope = 0 using Student’s *t*-tests; and by testing the significance of the Pearson’s correlation coefficient. However, the linear regression and the Pearson’s correlation coefficient detect only linear dependence; do not detect nonlinear or nonmonotone dependencies [63]. Therefore, we used distance correlation, distance covariance, and distance covariance test of independence [63,64] to address possible nonlinear dependencies between the variables under study. Distance correlation is a new dependence coefficient that measures all types of dependence between random vectors X and Y in arbitrary dimension [63]. Therefore, the distance covariance test of independence detects any nonlinear and nonmonotone dependence between two random variables [63]. We examined the relationship of R/S to DBH, TH, AGB, BGB, and total biomass by the same procedures. All analyses were performed at the 5% significance level using Microsoft Excel Data Analysis Tools and using the “Energy” package [65] in R [66].

Further, the default IPPC aboveground BEF (*BEF*_*h(IPCC)*_) for tropical braodleaf species and the respective BEF-based biomass density (*W*_*h(IPCC)*_) (computed using the default BEF and our estimated volume) were compared with the aboveground BEF from this study and the respective BEF-based biomass density. As for the respective uncertainties.

The default BEF-based biomass density is computed as follows (Eq. 20):

20 $$ {W}_{h(IPCC)}= BE{F}_{h(IPCC)}\times {V}_1\kern0.5em \left[{\mathrm{Mg}}^{2\kern0.5em }{\mathrm{ha}}^{\hbox{--} 1}\right] $$

The *BEF*_*h(IPCC)*_ and *V*_*1*_ are obtained from independent samples (separate surveys), therefore, the uncertainty (percent SE) of *W*_*h(IPCC)*_ can be computed as in Eq. 21 [30,31,39,58,59,62,67]:

21 $$ SE\%=\sqrt{SE{\%}_{BE{F}_{h(IPCC)}}^2+SE{\%}_{V_1}^2}\left[\%\right] $$

where $S{E}_{BE{F}_{h(IPCC)}}^2S{E}_{V_1}^2$ are percent standard errors associated with *BEF*_*h(IPCC)*_ and *V*_*1*_, respectively.

## Abbreviations

AGB: Aboveground biomass
BEF: Biomass expansion factor
BGB: Belowground biomass
DBH: Diameter at breast height
R/S: Root-to-shoot ratio
